# Supplementary material for: Changes in the Lifestyle of the Spanish University Population during Confinement for COVID-19
Source: Int J Environ Res Public Health. 2022 Feb 15;19(4):2210. doi: 10.3390/ijerph19042210 (PMC8872173; doi:10.3390/ijerph19042210)
Supplement: Supplementary file 1 [file ijerph-19-02210-s001.zip › ijerph-1513217-supplementary.pdf]

Table S1. Sociodemographic characteristics University Population.

		Total N=168 (100%)
Gender		
	Women	112 (66.7%)
	Men	56 (33.3%)
Birthplace		
	Spain	133 (79.2%)
	Latin America	28 (16.7%)
	Europe	4 (2.4%)
	Others	3 (1.8%)
Role		
	Students	12 (76.8%)
	R.P [1]	25 (14.9%)
	A.S [2]	14(8.3%)
Place of Residence		
	Family home	128 (76.2%)
	Shared flat	19 (11.3%)
	Student residence	10 (6%)
	Alone	11 (6.5%)
BMI (kg/m^2) [3]		
	Underweight (<18.5kg/m^2)	12 (7.1%)
	Normal weight (18.5-24.9kg/m2)	111 (66.1%)
	Pre-obesity (25-29.9kg/m2)	36 (21.4%)
	Obesity class I (30-34.9kg/m2)	8 (4.8%)
	Obesity class II (35-39.9kg/m2)	1 (0.6%)

[1] R.P: Research Professors. [2] A.S: Administrative Staff. [3] BMI: Body Max Index, expressed in kg/m2, using WHO classification.

Table S2. Distribution of students for studies and faculties.

		Total N=129 (100%)
Faculty		
	Health sciences	85 (65.9%)
	Social and humanity sciences	35 (27.1%)
	Higher polytechnic school	9 (7%)
Degree Program		
	Phycology	34 (26.4%)
	Human Nutrition and Dietetics	25 (19.4%)
	Sport and exercise sciences	12 (9.3%)
	Food Sciences and Technology	10 (7.8%)
	Translation and interpretation	9 (7%)
	Advertising and Public relations	8 (6.2%)
	Business Administration and Management	7 (5.4%)
	Industrial Organisation Engineering	5 (3.9%)
	Journalism	5 (3.9%)
	Audiovisual Communication	4 (3.1%)
	Agro-Food Engineering	4 (3.1%)
	Master General Health Psychology	4 (3.1%)
	Applied Languages	2 (1.6%)

Table S3. Dietary and lifestyle adaptations during the confinement, compared by Students vs. UE.

	Total N =168 (100%; CI)	Students N= 129 (76.8%; CI)	UE [1] N=39 (23.2%; CI)	*p*-Value [2]
How many times do you do the grocery shopping per week?				
1 or less per week	129 (76.8%; 69.6-82.9)	98 (76%; 67.6-83.0)	31 (79.5%; 63.5-90.7)	0.648
2 or more per week	39 (23.2%; 17.0-30.3)	31 (24%; 16.9-32.3)	8 (20.5%; 9.2-36.4)	
During the confinement, the consumption of fruits?				
Has increased	72 (42.9%; 35.2-50.7)	59 (45.7%; 36.9-54.7)	13 (33.3%; 19.0-50.2)	0.250
Has decreased	20 (11.9%; 7.4-17.7)	13 (10.1%; 5.4-16.6)	7 (17.9%; 7.5-33.5)	
Has stay the same	76 (45.2%; 37.5-53.0)	57 (44.2%; 35.4-53.1)	19 (48.7%; 32.4-65.2)	
During the confinement, the consumption of vegetables?				
Has increased	69 (41.1%; 33.5-48.9)	59 (45.7%; 36.9-54.7)	10 (25.6%; 13.0-42.1)	0.062
Has decreased	14 (8.3%; 4.6-13.5)	11 (8.5%; 4.3-14.7)	3 (7.7%; 1.6-20.8)	
Has stay the same	85 (50.6%; 42.7-58.3)	59 (45.7%; 36.9-54.7)	26 (66.7%; 49.7-80.9)	
During the confinement, the consumption of dairy products?				
Has increased	43 (25.6%; 19.1-32.8)	35 (27.1%; 19.6-35.6)	8 (20.5%; 9.2-36.4)	**<0.05**
Has decreased	21 (12.5%; 7.9-18.4)	20 (15.5%; 9.7-22.9)	1 (2.6%; 0.0-13.4)	
Has stay the same	104 (61.9%; 54.1-69.2)	74 (57.4%; 48.3-66.0)	30 (76.9%; 60.6-88.8)	
During the confinement, the consumption of pulses?				
Has increased	45 (26.8%; 20.2-34.1)	37 (28.7%; 21.0-37.3)	8 (20.5%; 9.2-36.4)	0.354
Has decreased	14 (8.3%; 4.6-13.5)	12 (9.3%; 4.9-15.6)	2 (5.1%; 0.6-17.3)	
Has stay the same	109 (64.9%; 57.1-72.0)	80 (62%; 53.0-70.4)	29 (74.4%; 57.8-86.9)	
During the confinement, the consumption of fish/seafood?				
Has increased	39 (23.2%; 17.0-30.4)	33 (25.6%; 18.3-34.0)	6 (15.4%; 5.8-30.5)	0.166
Has decreased	32 (19%; 13.4-25.8)	21 (16.3%; 10.3-23.8)	11 (28.2%; 15.0-44.8)	
Has stay the same	97 (57.7%; 49.8-65.3)	75 (58.1%; 49.1-66.7)	22 (56.4%; 39.6-72.1)	
During the confinement, the consumption of white meat?				
Has increased	28 (16.7%; 11.3-23.1)	25 (19.4%; 12.9-27.2)	3 (7.7%; 1.6-20.8)	0.208
Has decreased	10 (6%; 2.8-10.6)	8 (6.2%; 2.7-11.8)	2 (5.1%; 0.6-17.3)	

Has stay the same	130 (77.4%; 70.3-83.4)	96 (74.4%; 65.9-81.6)	34 (87.2%; 72.5-95.7)	
During the confinement, the consumption of red and processed meat?				
Has increased	15 (8.9%; 5.0-14.2)	13 (10.1%; 5.4-16.6)	2 (5.1%; 0.6-17.3)	0.243
Has decreased	33 (19.6%; 13.9-26.4)	28 (21.7%; 14.9-29.8)	5 (12.8%; 4.2-27.4)	
Has stay the same	120 (71.4%; 63.9-78.1)	88 (68.2%; 59.4-76.1)	32 (82.1%; 66.4-92.4)	
During the confinement, the consumption of pastries and snacks?				
Has increased	36 (21.4%; 15.4-28.4)	31 (24%; 16.9-32.3)	5 (12.8%; 4.2-27.4)	<0.01
Has decreased	51 (30.4%; 23.5-37.9)	45 (34.9%; 26.7-43.7)	6 (15.4%; 5.8-30.5)	
Has stay the same	81 (48.2%; 40.4-56.0)	53 (41.1%; 32.5-50.0)	28 (71.8%; 55.1-84.9)	
During the confinement, the consumption of rice/pasta/potatoes?				
Has increased	36 (21.4%; 15.4-28.4)	31 (24%; 16.9-32.3)	5 (12.8%; 4.2-27.4)	0.183
Has decreased	14 (8.3%; 4.6-13.5)	12 (9.3%; 4.9-15.6)	2 (5.1%; 0.6-17.3)	
Has stay the same	118 (70.2%; 62.7-77.0)	86 (66.7%; 57.8-74.7)	32 (82.1%; 66.4-92.4)	
During the confinement, the consumption of nuts?				
Has increased	27 (16.1%; 10.8-22.5)	23 (17.8%; 11.6-25.5)	4 (10.3%; 2.8-24.2)	0.215
Has decreased	36 (21.4%; 15.4-28.4)	30 (23.3%; 16.2-31.5)	6 (15.4%; 5.8-30.5)	
Has stay the same	105 (62.5%; 54.7-69.8)	76 (58.9%; 49.9-67.4)	29 (74.4%; 57.8-86.9)	
During the confinement, the consumption of low alcohol drinks (wine and beer)?				
Has increased	18 (10.7%; 6.4-16.4)	9 (7%; 3.2-12.8)	9 (23%; 11.1-39.3)	<0.001
Has decreased	101 (60.1%; 52.2-67.5)	87 (67.4%; 58.6-75.4)	14 (35.9%; 21.2-52.8)	
Has stay the same	49 (29.2%; 22.4-36.6)	33 (25.6%; 18.3-34.0)	16 (41%; 25.5-57.9)	
During the confinement, the consumption of spirits (vodka, gin, rum)?				
Has increased	2 (1.2%; 0.1-4.2)	2 (1.6%; 0.1-5.4)	0 (0%)	0.222
Has decreased	126 (75%; 67.7-81.3)	100 (77.5%; 69.3-84.3)	26 (66.7%; 49.7-80.9)	
Has stay the same	40 (23.8%; 17.5-30.9)	27 (20.9%; 14.2-28.9)	13 (33.3%; 19.0-50.2)	
During the confinement, the consumption of sugary drinks?				

Has increased	14 (8.3%; 4.6-13.5)	11 (8.5%; 4.3-14.7)	3 (7.7%; 1.6-20.8)	0.972
Has decreased	96 (57.1%; 49.2-64.7)	74 (57.4%; 48.3-66.0)	22 (56.4%; 39.6-72.1)	
Has stay the same	58 (34.5%; 27.3-42.2)	44 (34.1%; 25.9-42.9)	14 (35.9%; 21.2-52.8)	
Do you cook more often during the confinement?				
Yes	123 (73.2%; 65.8-79.7)	92 (71.3%; 62.6-78.9)	31 (79.5%; 63.5-90.7)	0.313
No	45 (26.8%; 20.2-34.1)	37 (28.7%; 21.0-37.3)	8 (20.5%; 9.2-36.4)	
Have you increased the number of meals these days?				
Has increased	43 (25.6%; 19.1-32.8)	31 (24%; 16.9-32.3)	12 (30.8%; 17.0-47.5)	0.182
Has decreased	45 (26.8%; 20.2-34.1)	39 (30.2%; 22.4-38.9)	6 (15.4%; 5.8-30.5)	
Has stay the same	80 (47.6%; 39.8-55.4)	59 (45.7%; 36.9-54.7)	21 (53.8%; 37.1-69.9)	
During the confinement, your physical activity?				
Has increased	61 (36.3%; 29.0-44.0)	52 (40.3%; 31.7-49.3)	9 (23.1%; 11.1-39.3)	<0.05
Has decreased	83 (49.4%; 41.6-57.2)	57 (44.2%; 35.4-53.1)	26 (66.7%; 49.7-80.9)	
Has stay the same	24 (14.3%; 9.3-20.5)	20 (15.5%; 9.7-22.9)	4 (10.3%; 2.8-24.2)	

[1] UE: University Employees. [2] Differences between role were evaluated by the Pearson Chi square test ($p < 0.05$).
